# Special Issue “Recent Synthetic Aspects on the Chemistry of Nitro, Nitroso and Amino Compounds”

**DOI:** 10.3390/molecules22010009

**Published:** 2016-12-23

**Authors:** Alessandro Palmieri

**Affiliations:** Green Chemistry Group, School of Sciences and Technology, Chemistry Division, University of Camerino, via S. Agostino 1, 62032 Camerino (MC), Italy; Alessandro.palmieri@unicam.it; Tel.: +39-0737-402262

**Keywords:** amines, flow chemistry, nitro compounds, β-nitroacrilates, *aza*-Michael reaction, leishmaniasis, quaternary ammonium salts, formilations, peptides, organocatalysis

## Abstract

Nitrogen-containing molecules are key scaffolds that are widely applied in organic synthesis as precursors of highly functionalized materials, and are also investigated for their biological activities. This Special Issue collects seven innovative contributions which expand our knowledge of the chemistry of nitro compounds, amines, diazonium salts, and peptides, and that provide a good overview about their main reactivities.

This Special Issue collects seven original research articles focused on the chemistry of nitro, nitroso, and amino derivatives. In particular, these studies were directed towards the implementation of innovative synthetic protocols for the generation of new carbon-carbon and carbon-heteroatom bonds, and towards preparing and evaluating new biologically active derivatives. In this context, Baxendale et al. presented a series of flow-based protocols aimed at preparing diazonium salts, starting from aryl amines, for subsequent in situ utilization [1]. Their studies deal with the evaluation of different procedures for the aryl diazonium salts’ formation compatibly with the flow chemical conditions, and the implementation of protocols depending on the phase used (aqueous, organic, and solid phase). With this approach, the authors disclosed new synthetic pathways amenable to avoiding the isolation and handling of diazonium species, reducing the potential risks due to the health hazards associated with such compounds. Bosica and Abdilla reported a new heterogeneous *aza*-Michael addition of primary aliphatic and aromatic amines to a variety of electron-poor alkenes [2]. In particular, they demonstrated that acidic alumina can be used, under solvent-free conditions, to selectively synthesize the mono-adduct derivatives in excellent yields, overcoming the typical formation of bis-adduct byproducts. Robledo and co-workers exploited the nucleophilicity of amines to prepare halomethylated quaternary ammonium salts, non-halogenated quaternary ammonium salts, and halomethylated choline analogs [3]. These compounds were successively investigated to assess their in vitro antileishmanial activity in axenic amastigotes of L. *(Viannia) panamensis* (MTT, 3-(4,5-dimethylthiazol-2-yl)-2,5-diphenyl-tetrazolium bromide, micromethod) and in intracellular amastigotes of *L. (V) panamensis* (flow cytometry), and their cytotoxicity in human promonocytic cells U-937. The reactivity of the amines was also studied by Tornesello et al. for the formylation of *N*-terminus peptides [4]. In particular, they developed a new solid-phase synthetic protocol, which involves the use of formic acid, DCC (*N*,*N*-dicyclohexylcarbodiimmide) in the presence of chloroform, and that affords high quantities of *N*-formylated peptides, allowing the facile removal of side products by resin washing. The usefulness of the procedure was tested on two chemotactic hexapetides, namely Met1-Leu2-Lys3-Leu4-Ile5-Val6 and Met1-Met2-Tyr3-Ala4-Leu5-Phe6. In a further research article, Bernardi and Fochi extended their previous studies to implement a general organocatalyzed enantioselective transfer hydrogenation reaction of β,β-disubstituted nitroalkenes for producing optically active nitroalkanes [5]. This transformation was carried out using *tert*-butyl Hantzsch ester in the presence of a simple and commercially available Jacobsen type thiourea catalyst. Products were obtained in excellent yields and enantiomeric excess, and additional kinetic studies allowed the proposal of a plausible reaction transition state model. Fioravanti et al. described a very interesting study concerning the direct comparison between the C-CF_3_ and C-CH_3_ substituted *N*-protected aldimines in *aza*-Henry addition reactions [6]. In this context, C-alkyl aldimines easily reacted with nitroalkanes under catalyst-free and solvent-free conditions, vice versa, C-CF_3_ aldimines produced the corresponding products only in the presence of ZrCl_4_. In general, good yields and diastereomeric ratios (dr) were observed in all cases, particularly for aldimines endowed with a significant steric hindrance, which afforded the expected adducts a dr of 99:1. Finally, we developed a new one-pot synthesis of quinoline-2-carboxylates starting from β-nitroacrylates and 2-aminobenzaldehydes [7]. The protocol involves two steps: the first step is performed under promoter-free and solvent-free conditions, and it consists of an *aza*-Michael addition and an intramolecular Henry reaction. The second step entails the elimination of a molecule of water and nitrous acid, and requires the addition of solvent and base (supported BEMP, 2-*tert*-Butylimino-2-diethylamino-1,3-dimethylperhydro-1,3,2-diazaphosphorine). The products were generally obtained with moderate to good overall yields and the use of heterogeneous conditions avoided any complex aqueous work-up, with evident advantages from a sustainable point of view.

## References

[1] Hu T., Baxendale I.R., Baumann M. (2016). Exploring Flow Procedures for Diazonium Formation. Molecules.

[2] Bosica G., Abdilla R. (2016). Aza-Michael Mono-addition Using Acidic Alumina under Solventless Conditions. Molecules.

[3] Duque-Benítez S.M., Ríos-Vásquez L.A., Ocampo-Cardona R., Cedeño D.L., Jones M.A., Vélez I.D., Robledo S.M. (2016). Synthesis of Novel Quaternary Ammonium Salts and Their In Vitro Antileishmanial Activity and U-937 Cell Cytotoxicity. Molecules.

[4] Tornesello A.L., Sanseverino M., Buonaguro F.M. (2016). Solid Phase Formylation of N-Terminus Peptides. Molecules.

[5] Bernardi L., Fochi M. (2016). A General Catalytic Enantioselective Transfer Hydrogenation Reaction of β,β-Disubstituted Nitroalkenes Promoted by a Simple Organocatalyst. Molecules.

[6] Pelagalli A., Pellacani L., Scandozza E., Fioravanti S. (2016). Aza-Henry Reactions on C-Alkyl Substituted Aldimines. Molecules.

[7] Gabrielli S., Giardinieri A., Sampaolesi S., Ballini R., Palmieri A. (2016). A New One-Pot Synthesis of Quinoline-2-carboxylates under Heterogeneous Conditions. Molecules.

